# A Halocin Promotes DNA Uptake in *Haloferax mediterranei*

**DOI:** 10.3389/fmicb.2019.01960

**Published:** 2019-09-18

**Authors:** Shaoxing Chen, Siqi Sun, Gregory A. Korfanty, Jingwen Liu, Hua Xiang

**Affiliations:** ^1^Anhui Provincial Key Laboratory of the Conservation and Exploitation of Biological Resources, College of Life Sciences, Anhui Normal University, Wuhu, China; ^2^State Key Laboratory of Microbial Resources, Institute of Microbiology, Chinese Academy of Sciences, Beijing, China; ^3^Department of Biology, McMaster University, Hamilton, ON, Canada; ^4^College of Life Sciences, University of Chinese Academy of Sciences, Beijing, China

**Keywords:** haloarchaea, halophilic archaea, DNA uptake, natural transformation, halocin, archaeocin

## Abstract

Halocins are antimicrobial peptides or proteins that are produced by halophilic archaea. Although their function in inhibiting the growth of closely related haloarchaeal strains is well known, other physiological functions of halocins have also been proposed in recent years. To unveil the possible function and mechanism of halocins in DNA uptake, the halocin H4 producing strain *Haloferax mediterranei* DF50-ΔEPS (incapable of EPS production) was used in this study. We found that deletion of the *halH4* resulted in the strain DF50-ΔEPSΔ*halH4* which exhibited loss of natural DNA uptake ability. Moreover, supernatants of the halocin producing strain were capable of inducing the ability to uptake DNA. Obviously, halocin is likely responsible for inducing DNA uptake. Cell surface ultrastructures of these strains are varied from strains DF50-ΔEPS to DF50-ΔEPSΔ*halH4*. The cell surface of strain DF50-ΔEPS is rough due to numerous pinholes, while that of the strain DF50-ΔEPSΔ*halH4* is smooth without visible pinholes. The morphology of the *halH4* complemented strain, DF50-ΔEPSΔ*halH4::H4*, shows an intermediate phenotype between strains DF50-ΔEPS and DF50-ΔEPSΔ*halH4*. We speculate that halocin H4 may accelerate DNA uptake by perforating the cell surface ultrastructure. The halocin H4 may represent a novel inducer or activator of DNA uptake in *Hfx. mediterranei*.

## Importance

Halocin H4 (HalH4) secreted by *Haloferax mediterranei* has always been taken as a typical antimicrobial protein. In this work, we found that deletion of the halocin H4-encoding gene, *halH4*, blocks the DNA uptake in *Hfx. mediterranei* by changing the cell surface ultrastructure. It is the first study on the function of halocins in natural environments, substantially contributing to new knowledge in this domain.

## Introduction

Archaeocins are antimicrobial peptides or proteins produced by archaea, currently including the halocins produced by halophilic archaea as well as sulfolobicins by *Sulfolobus* spp. (O’Connor and Shand, 2002). Halocins were initially discovered during a survey of antagonistic interactions among different members of the class *Halobacteria* (Rodriguez-Valera et al., 1982). To date, at least 11 halocins have been reported, i.e., HalHA1, HalHA3, HalA4, HalH1, HalH4, HalH6/H7, HalR1, HalSech7A, HalSH10, HalS8, and HalC8 (O’Connor and Shand, 2002; Li et al., 2003; Pasić et al., 2008; Karthikeyan et al., 2013; Besse et al., 2015; Kumar et al., 2016; Kumar and Tiwari, 2017; Mazguene et al., 2017). Among these halocins, the genes encoding H6/H7, H4, S8, and C8 have been described and analyzed in depth as well (Meseguer et al., 1995; Cheung et al., 1997; Sun et al., 2005).

Although halocin H6/H7 produced by *Haloferax gibbonsii* has been reported to act on the Na^+^/H^+^ antiporter (Meseguer et al., 1995), it has also been reported that halocins H4 and C8 can morphologically change rod cells into spherical cells in sensitive strains, which ultimately leads to cell lysis (Meseguer and Rodriguez-Valera, 1986; Li et al., 2003). The mechanisms of action on the target strain of other halocins have not yet been unveiled. Moreover, several studies have shown that the antagonistic activity of haloarchaea could be attributed to the production of halocins (or other antimicrobial agents) (Oren, 1994; Ghanmi et al., 2016; Quadri et al., 2016). Nevertheless, no antihaloarchaeal substances are present within a natural hypersaline environment, where some halocin-producing strains survive. The physiological and ecological importance of halocins in hypersaline environments and in competition for nutrients and space remains elusive (Kis-Papo and Oren, 2000; Oren and Hallsworth, 2014). Therefore, it would be interesting to investigate other possible functions of halocins in the environment, e.g., their involvement in genetic exchange or natural transformation.

The genetic exchange between *Haloferax volcanii* (formerly *Halobacterium volcanii*) cells has previously been reported (Mevarech and Werczberger, 1985). Such horizontal gene transfer (HGT) events can deliver genes from the donor cells to the recipient cells. Large scale genomic DNA transfer and recombination between the *Hfx. volcanii* and *Hfx. mediterranei* cells based on cell fusion has also recently been reported (Naor et al., 2012; Naor and Gophna, 2013). HGT plays an important role in speciation, niche adaptation and species diversity maintenance in archaea and bacteria (Papke et al., 2015; Koonin, 2016; Wagner et al., 2017). Natural transformation, an important pathway of HGT, has been reported in over 80 bacterial species (e.g., *Helicobacter pylori*, *Campylobacter jejuni*, *Acinetobacter baumannii*, *Ralstonia solanacearum*, and *Agrobacterium tumefaciens*) (Johnston et al., 2014) and in some haloarchaeal species (Chen et al., 2012).

The prerequisite for natural DNA uptake is the development of a natural competence state or formation of a special channel. Many proteins have been identified as inducers and/or regulators involved in this process in bacteria, for instance, ComK in *Bacillus subtilis* (Mohan and Dubnau, 1990), Sxy in *Haemophilus influenza* (Lo Scrudato et al., 2014), and TfoX and QstR in *Vibrio cholerae* (Borgeaud et al., 2015). The Ced system, a DNA uptake system, is exclusive to microorganisms in Crenarchaeota phylum (van Wolferen et al., 2016). This system contains two principal proteins, CedA, a membrane protein resembling ComEC of bacterial competence systems, and CedB, a membrane-bound protein which exhibits ATPase activity essential for DNA transfer (van Wolferen et al., 2016). In halophilic archaea, such as *Natrialba magadii*, it has been reported that bacitracin, an antimicrobial polypeptide produced by bacteria (Ouyang et al., 2010), can be used to induce the formation of spheroplasts and to promote DNA uptake (Mayrhofer-Iro et al., 2013). However, the involvement of halocins (either produced by the cell itself or utilized from environmental sources) in DNA uptake has never been investigated in halophilic archaea.

In this study, we focused on the involvement of halocin H4 in DNA uptake in *Hfx. mediterranei*. Initially, cells of *Hfx. mediterranei* strain DF50 were used as the recipients to test the ability to uptake DNA, but very few transformants were observed. Exopolysaccharide deficient strains were used to avoid the blocking effect of the exopolysaccharides on DNA uptake (Wang et al., 2011). Thus, *Hfx. mediterranei* strain DF50-ΔEPS (incapable of producing exopolysaccharide) and DF50-ΔEPSΔ*halH4* (incapable of producing exopolysaccharide and halocin H4) were applied to explore the difference of their DNA uptake abilities. Furthermore, halocin(s) produced by strain *Haloferax* sp. Q22 was used to treat cells of strain DF50-ΔEPSΔ*halH4* before conducting the corresponding transformation to identify the promotion of halocin in DNA uptake. In addition, cell surface ultrastructures of strains DF50, DF50-ΔEPS, DF50-ΔEPSΔ*halH4*, DF50-ΔEPSΔ*halH4::H4* (complemented strain to the *halH4* mutated strain), and DF50-ΔEPSΔ*halH4* treated with halocin(s) produced by strain *Haloferax* sp. Q22 were also analyzed using scanning electron microscopy. This is the first attempt to probe the function of halocin in relation to inducing the DNA uptake in *Hfx. mediterranei*.

## Materials and Methods

### Strains, Culture Conditions, and Plasmids

Strains and plasmids used in this study are listed in Table 1. The oligonucleotides used in this study are listed in Table 2. Strains of *Escherichia coli* were cultivated in lysogeny broth (LB) at 37°C with ampicillin at a final concentration of 100 μg ml^–1^ if necessary (Sambrook and Russell, 2001). *E. coli* JM109 was used as the host strain for the construction of recombinant plasmids (Sambrook and Russell, 2001). Plasmids used for transforming haloarchaeal cells were shuttled into *E. coli* JM110 (*dam*^–^ and *dcm*^–^) (Palmer and Marinus, 1994). In this study, *Hfx. mediterranei* strains DF50 and DF50-ΔEPS were provided by Zhao et al. (2013). The *halH4* deletion mutant of the strain DF50-ΔEPS was named stain DF50-ΔEPSΔ*halH4*. Strains DF50, DF50-ΔEPS and DF50-ΔEPSΔ*halH4* were cultivated in AS-168 medium supplemented with 50 μg ml^–1^ uracil at 37°C for 7 days (shaking at 180 rpm) (Liu et al., 2011). Strains *Haloferax* sp. Q22 and DF50-ΔEPSΔ*halH4::H4* were cultivated in AS-168 medium (Liu et al., 2011). AS-168SY medium derived from AS-168 medium by omitting the yeast extract was used to screen cells that contained the active *pyrF* gene (Liu et al., 2011).

**TABLE 1:** Strains and plasmids used in this study.

**Strains and plasmids**	**Description**	**Source or reference**
**Strains**		
*Haloferax* sp. strain Q22	Wild type; halocin producing strain	Chen et al., 2016
*Hfx. mediterranei* strain DF50	The *pyr*F gene deletion mutant of *Hfx. meditrranei* ATCC33500	Liu et al., 2011
*Hfx. mediterranei* strain DF50-ΔEPS	The *eps* gene deletion mutant of *Hfx. mediterranei* DF50	Zhao et al., 2013
*Hfx. mediterranei* strain DF50-ΔEPSΔ*halH4*	The *hal*H4 deletion mutant of *Hfx. mediterranei* strain EPS	This study
*Hfx. mediterranei* strain DF50-ΔEPSΔ*halH4::H4*	Strain EPSH containing a recombinant plasmid, pWH4; *hal*H4^+^; *pyr*F^+^	This study
*E. coli* JM 109	Widely used host strain for molecular cloning, *rec*A1, *end*A1, *gyr*A96, *thi*^–^, *hsd*R17, *sup*E44, *rel*A1	This study
*E. coli* JM 110	The *dam*^–^ and *dcm*^–^ of *E. coli* JM 109	TaKaRa, Japan
**Plasmids**		
pMD-18T	2.7 kb, cloning T-vector; Amp^r^	TaKaRa, Japan
pHFX	4.0 kb, lacking the origin for the replication in haloarchaea; gene knockout vector; Amp^r^	Liu et al., 2011
pHFX-UDH4	The up (521 bp) and down (526 bp) fragments of *hal*H4 were combined together and inserted into plasmid pHFX at the multiple cloning site for the gene knockout of *hal*H4 gene	This study
pWL502	7.9 kb, shuttle vector with *pyr*F marker; Amp^r^	Liu et al., 2011
pWH4	9.0 kb; derivative of pWL502 containing *hal*H4 gene and its native promoter	This study

**TABLE 2:** Oligonucleotides used in this study.

**Name**	**Sequence (5′-3′)**	**Description**
UPH4F1	GTTATCATATTCTTCGGTAG	For the construction of the gene knockout plasmid pHFX-UDH4
UPH4R1	ACAGACGGACGAGTAACACTTCCCGAATGTGACTCGTGAT	
DWH4F2	ACATTCGGGAAGTGTTACTCGTCCGTCTGTAGCGGTGCCT	
DWH4R2	ATGGGTGGTGGACTGCAGCG	
H4F	ATTACACCGACTTTGCGCTC	For the detection of the *hal*H4 gene
H4R	GCAACGTACACCATCTCGTC	
H4CF	CG*GGTACC*TAGATGTCGAAAGACAGAGATGG	For the amplification of the complete gene of *hal*H4
H4CR	CG*GGATCC*CTGTTTCCTACTCCGTTGTT	

*The restriction sites of *Kpn*I (*GGTACC*) and *Bam*HI (*GGATCC*) were italic.*

### Construction of *halH4* Gene Deletion Mutant

Primers listed in Table 2 were designed by the online software Primer3web version 4.1.0^1^. To construct the gene knockout plasmid, a pair of primers (UPH4F1/UPH4R1) was used to amplify upstream DNA fragments of the *halH4* gene from *Hfx. mediterranei* strain DF50-ΔEPS (Table 1) by PCR amplification. Similarly, the primer pair DWH4F2/DWH4R2 was designed to obtain the downstream fragments of the *halH4* gene. PCR amplification was performed in a 50 μl reaction mixture composed of 25 μl 2 × Fast Taq PCR MasterMix (BioMed, China), 2 μl each forward and reverse primers (10 μm), 1 μl template DNA (∼100 ng/μl), and 20 μl ddH_2_O. The PCR cycling conditions included an initial denaturation step (5 min, 94°C) followed by 30 cycles of denaturation (1 min, 94°C), annealing (1 min, 53°C), and extension (1 min, 72°C) and a final extension period (5 min, 72°C). A 521-bp DNA fragment (F521) directly upstream and a 526-bp DNA fragment (F526) directly downstream of the *halH4* (HFX_5264) gene were amplified using the primer pairs UPH4F1/UPH4R1 and DWH4F2/DWH4R2, respectively. These two DNA fragments were purified with a DNA extraction kit (Axygen, United States), and then used as the templates for overlapping extension PCR amplification. The overlapping PCR reaction mixture (50 μl) was composed of 25 μl 2 × Fast Taq PCR Master Mix (BioMed, China), 2 μl each forward (UPH4F1) and reverse (DWH4R2) primers, 1 μl chromosomal DNA (∼100 ng/μl), 2 μl each purified F521 and F526, and 16 μl ddH_2_O. The PCR cycling conditions were the same as described above. The PCR products were purified with a DNA extraction kit (Axygen, United States) in accordance with the manufacturer’s instructions. The purified PCR products were inserted into pMD-18T (TaKaRa, Japan) with the T-A cloning strategy in *E. coli* JM 109 (Sambrook and Russell, 2001). After verification by sequencing, the recombinant plasmids were extracted with a plasmid extraction kit (Axygen, United States) and digested with *Bam*HI plus *Kpn*I (New England Biolabs, United States). The purified DNA fragment was inserted into the plasmid pHFX at the cohesive sites of *Bam*HI and *Kpn*I, resulting in the gene knockout plasmid pDH4. Plasmid pDH4 was verified by PCR amplification and DNA sequencing prior to transformation. Plasmid pDH4 was shuttled into *E. coli* JM110 and harvested for polyethylene glycol (PEG)-mediated transformation of the haloarchaeal DF50-ΔEPS strain. The PEG-mediated transformation was performed according to the method described by Cline et al. (1989). The above process regarding the *pyrF*-based gene knockout in *Hfx. mediterranei* was followed in accordance with the method described by Liu et al. (2011). To obtain the *halH4* deletion mutants, transformants picked from the selective plates were spotted onto new plates with sterile toothpicks, and then the rest of the cells at the tip were re-suspended in 20 μl sterile distilled water. The supernatants of the lysates (2 μl), after centrifugation at 12,000 *g* for 3 min, were taken as a PCR template and the primer pair H4F and H4R (Table 2) were used to screen the DNA of the transformants. The *halH4* deletion mutant was named *Hfx. mediterranei* strain DF50-ΔEPSΔ*halH4* abbreviated to strain DF50-ΔEPSΔ*halH4*. The resultant strain was verified by PCR amplification.

### Construction of *halH4* Deletion Mutant Complementary Strain

To construct the complementary strain of the *halH4* deletion mutant (strain DF50-ΔEPSΔ*halH4*), the complete *halH4* gene, which was amplified with the primer pair H4CF and H4CR (Table 2), was inserted into the expression shuttle vector pWL502 (Cai et al., 2012) derived from plasmid pWL102 (Lam and Doolittle, 1989) at the restriction sites of *Kpn*I and *Bam*HI resulting in the complementary plasmid pWH4. Then, the recombinant plasmid pWH4 was introduced into DF50-ΔEPSΔ*halH4* cells via the PEG-mediated transformation approach (Cline et al., 1989) resulting in the complementary strain DF50-ΔEPSΔ*halH4*::*H4*. The resultant complementary strain was verified by PCR amplification and DNA sequencing.

### Determination of the DNA Uptake Efficiency

To determine the DNA uptake efficiency of strains DF50, DF50-ΔEPS, and DF50-ΔEPSΔ*halH4*, shuttle vector pWL502 (Cai et al., 2012) was used. Plasmid pWL502 harbors two replicons which confers its replication in *E. coli* and haloarchaea. The complete *pyrF* gene on plasmid pWL502 can complement the *pyrF* deletion mutants, e.g., DF50, DF50-ΔEPS, and DF50-ΔEPSΔ*halH4* strains (Table 1). One hundred μl cell suspension of DF50-ΔEPS and DF50-ΔEPSΔ*halH4* strains was inoculated into liquid AS-168 medium supplemented with uracil (50 μg ml^–1^) for cultivation (37°C, 180 rpm). When the optical density at 600 nm reached 1.0, 1.5 ml cell suspension of strains DF50-ΔEPS and DF50-ΔEPSΔ*halH4* in the late exponential phase were harvested and washed three times with 5% (w/v) sterile NaCl solution (1 ml). Cells were re-suspended in the above 5% (w/v) NaCl solution and then gently mixed with plasmid pWL502 (∼6 μg dsDNA/1 ml cell suspension). Mixtures were then allowed to sit at room temperature for 1 h and were subsequently spread onto AS-168SY agar plates (1 ml for each plate) (Liu et al., 2011). Taking the simple transformation method from Chen et al. (2012), the culture medium and selection marker procedure was modified to fit the growth of strains DF50-ΔEPS and DF50-ΔEPSΔ*halH4* and other operations were left unchanged. This process of transformation is referred to as the simulation of natural transformation (Chen et al., 2012). Before transformation in haloarchaeal cells, plasmid pWL502 was shuttled into *E. coli* JM110 (*dam*^–^ and *dcm*^–^) to prevent DNA degradation by its native restriction-modification system. Three biological replicates were performed, and the mean number of colonies was calculated.

Numerous transformants were observed on the selective agar plates when cells of strain DF50-ΔEPS were used as recipients. Five transformants were picked arbitrarily and inoculated into liquid medium for plasmid propagation. After cultivation at 37°C for 7 days with shaking, plasmids were extracted from each culture by a plasmid extraction kit (Axygen, United States). The suspected plasmid was verified by transforming it back to *E. coli* JM109 and executing enzyme digestion with *Kpn*I (New England Biolabs, United States).

### Detection of Halocin(s) Produced by Strain *Haloferax* sp. Q22

Strain *Haloferax* sp. Q22 was isolated from the Yunnan salt mine. No haloarchaeal colonies grew surrounding the original colony of strain *Haloferax* sp. Q22. To test the inhibition effect of strain *Haloferax* sp. Q22 to other haloarchaeal strains, 20 strains from nine haloarchaeal genera were selected in this study (Supplementary Table S1). All the haloarchaeal strains listed in Supplementary Table S1 were cultured in liquid AS-168 medium (pH 7.5). Cell suspension (500 μl, OD_600_≈1.0) of each strain was spread onto AS-168 agar plate. A small round sterile filter paper (6 mm in diameter) was put onto the agar plate. Then, 10 μl cell suspension (OD_600_≈1.0) of strain *Haloferax* sp. Q22 were dropped onto the filter paper followed by cultivation at 37°C for 1 or 2 weeks, depending on the growth rate of the indicator strain.

To further determine whether the antagonistic effect was caused by the production of halocin, we harvested the supernatants of the strain *Haloferax* sp. Q22 in the stationary phase and used strain DF50-ΔEPSΔ*halH4* as the indicator. The indicating plates were constructed by mixing cells of strain DF50-ΔEPSΔ*halH4* in the exponential phase with culture medium in the ratio of 100:10 (culture medium: cells suspension, v/v) at 50°C. A sterile puncher was used to create holes of 6 mm in diameter on the indicator plates. The supernatants of the strain *Haloferax* sp. Q22 were prepared via centrifugation at 12,000 g for 3 min at 15°C followed by filtration with a membrane filter (pore diameter, 0.22 μm).

In order to determine whether the antagonistic effect of supernatants was caused by halocin(s), a proteinaceous substance, supernatants of the strain *Haloferax* sp. Q22 were treated with protease K at the final concentration of 5 mg/ml at 37°C for 2 h. Then, 100 μl of the protease K treated supernatants was poured into the holes on the indicator plate. Equal volume of an untreated supernatants and a solution of protease K (5 mg/ml) in liquid AS-168 medium were taken as the controls.

Another portion of supernatants were heated at 90°C for 10, 30, and 60 min, respectively. Then, 100 μl of each heat-treated samples were poured into the holes to explore the antagonistic activity. The supernatants without heat treatment were taken as a control.

### Determination of the General Properties of the Halocin(s) From Strain *Haloferax* sp. Q22

To determine the molecular size of the halocin(s) produced by strain Q22, the cell free supernatants were centrifuged with an ultrafiltration membrane with a molecular weight cut-off of 3,000 and 10,000 Da at 3,000 *g* (centrifugal force). After ultrafiltration, the inhibition activity of effluxes (100 μl) was analyzed with an indicator plate. Strain DF50-ΔEPSΔ*halH4* was used to construct the indicator plate.

To detect the desalting activity of the halocin(s), the solvent system of halocin(s) was changed to distilled water using ultrafiltration with a molecular weight cut-off of 3,000 Da at 3,000 *g* (centrifugal force) three times. The volume of retention decreased from 10 ml to 1 ml after ultrafiltration, then refilled to 10 ml with distilled water three times. One hundred microliters of the resultant retention was used for detection of inhibition activity on the same indicator plate as above.

### Promotion of Transformation Efficiency by Halocin(s)

To detect the effect of halocin(s) on promotion of DNA uptake, supernatants of strain *Haloferax* sp. Q22 were harvested for further study. After cultivation at 37°C for 10 days in liquid AS-168 medium, supernatants of strain *Haloferax* sp. Q22 were prepared via centrifugation at 12,000 *g* for 3 min at 15°C. Then, the supernatants were filtered with a membrane filter (pore diameter, 0.22 μm), and then concentrated to two, four, and tenfold (onefold equal to original), respectively, with tangential flow filtration (molecular weight cutoff, 10 kDa).

The original supernatants were used to determine the halocin(s) activity against strain DF50-ΔEPSΔ*halH4*. Different volumes (40, 60, 80, and 100 μl) of the original supernatants were poured into holes (6 mm in diameter) on the indicating plate. Strain DF50-ΔEPSΔ*halH4* was taken as the indicator. After that, the inoculated plates were cultivated at 37°C for 2 days for recording.

Cells of strain DF50-ΔEPSΔ*halH4* were harvested by centrifugation (12,000 *g* for 3 min), and then re-suspended with original (onefold), two, four, and tenfold condensed supernatants, respectively. After holding at room temperature for 1 h, the supernatants were then removed by centrifugation (12,000 *g* for 3 min). Then, simulation of natural transformation with recombinant plasmid pWL502 was conducted (Chen et al., 2012). Transformants were also verified by plasmid extraction and enzyme digestion.

### Morphological Observation of Cell Surface Ultrastructure

Strains DF50, DF50-ΔEPS, DF50-ΔEPSΔ*halH4*, and DF50-ΔEPSΔ*halH4::H4* were cultured in liquid AS-168 medium supplemented with uracil if necessary. Next, 1 ml of the cell suspension for each strain was inoculated into a 250 ml flask with 100 ml liquid medium for cultivation at 37°C for 4 days with shaking (180 rpm). Cells were harvested by centrifugation at 12,000 *g* for 3 min and washed three times with 1 ml sterile 10% (w/v) NaCl and ultimately re-suspended in this solution. In addition, cells of strain DF50-ΔEPSΔ*halH4* were harvested by centrifugation (12,000 *g* for 3 min) and re-suspended in twofold condensation of the supernatants of strain *Haloferax* sp. Q22 prior to performing the scanning electron microscope observation. Electron microscopy was conducted according to the procedure described by Muller et al. (2010) with a small modification at the step of cell fixing. Here, the haloarchaeal cells were fixed in 2% (v/v) glutaric dialdehyde (Sigma-Aldrich) in 1 ml sterile 10% (w/v) NaCl. The cell surface ultrastructure was observed by scanning electron microscopy (HITACHI SU8010, Japan) in accordance with the approach described by Muller et al. (2010).

## Results

### Construction of the *halH4* Deletion Mutant and Its Complementary Strain

It has been reported that production of exopolysaccharide (EPS, as an extracellular barrier) can block natural transformation in bacteria (Wang et al., 2011). Therefore, to explore the correlation between halocin H4 and DNA uptake, *Hfx. mediterranei* strain DF50-ΔEPS (incapable of EPS production, Zhao et al., 2013) was used as the parental strain. The *halH4* gene was deleted from strain DF50-ΔEPS resulting in strain DF50-ΔEPSΔ*halH4* (incapable of production of both EPS and halocin H4), which was verified by PCR amplification (Supplementary Figure S1). After verification via PCR (Supplementary Figure S1) and DNA sequencing (data not shown), the complementary strain of the *halH4* deletion mutant was successfully constructed.

The growth rates of strains DF50-ΔEPS and DF50-ΔEPSΔ*halH4* were similar when grown on AS-168 agar plates and in liquid medium supplemented with 50 μg ml^–1^ uracil, indicating the deletion of the *halH4* gene did not significantly impair their growth (Supplementary Figure S2).

After verification via PCR amplification and DNA sequencing, recombinant plasmid pWH4 was successfully introduced into strain DF50-ΔEPSΔ*halH4* resulting in the *halH4* deletion mutant’ complementary strain, DF50-ΔEPSΔ*halH4::H4* (Supplementary Figure S3).

### Plasmid DNA Uptake in Cells of Strains DF50, DF50-ΔEPS, and DF50-ΔEPSΔ*halH4*

**FIGURE 1:**
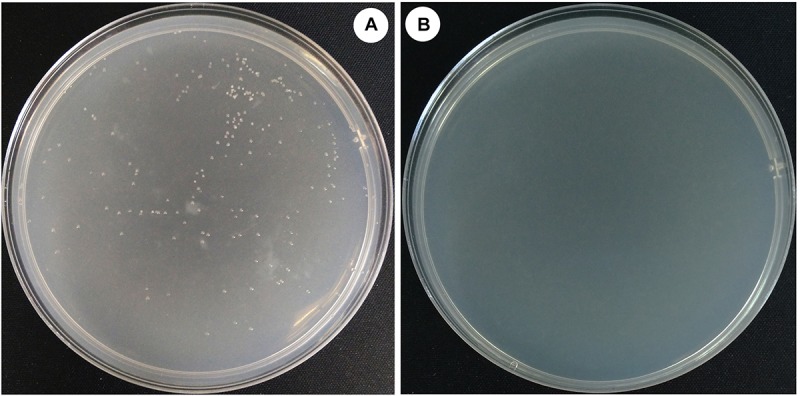
Efficiency of the DNA uptake in *Haloferax mediterranei*. Plasmid pWL502 was taken as external DNA to transform strains of *Hfx. mediterranei*. The procedure of simulation of natural transformation was described by Chen et al. (2012). Cells of strains DF50-ΔEPS **(A)** and DF50-ΔEPSΔ*halH4* **(B)** were used as recipients.

To evaluate the effect of the deletion of *halH4* on the DNA uptake in *Hfx. mediterranei*, a transformation was performed by using cells of strains DF50-ΔEPS and DF50-ΔEPSΔ*halH4* as recipients and pWL502 plasmid as extracellular DNA in accordance with the method described by Chen et al. (2012). Transformants were present for strain DF50-ΔEPS and absent for strains DF50 (data not shown) and DF50-ΔEPSΔ*halH4* (Figure 1). As such, EPS production significantly impeded the DNA uptake of strain DF50 (data not shown). Plasmid DNA uptake efficiency of the cells of strain DF50-ΔEPS was approximately 1.7 ± 0.3 × 10^3^ transformants/μg dsDNA (Table 3). Transformants were verified by plasmid extraction and enzyme digestion (data not shown). These experiments indicated that the cells of strain DF50-ΔEPS were accessible to DNA uptake. When the *halH4* gene was deleted, the DNA uptake capability of the cells of strain DF50-ΔEPSΔ*halH4* was lost completely.

**TABLE 3:** Influence of the *hal*H4 on DNA uptake efficiency.

**Strain**	**DNA uptake efficiency (×10^3^ transformants/μg dsDNA)**
*Hfx. mediterranei* strain DF50	0.003 ± 0.001
*Hfx. mediterranei* strain DF50-ΔEPS	1.7 ± 0.3
*Hfx. mediterranei* strain DF50-ΔEPSΔ*halH4*	0.002 ± 0.002

### Production and Properties of Halocin(s) Produced by Strain *Haloferax* sp. Q22

The antagonistic experiments showed that strain *Haloferax* sp. Q22 possessed a relatively wide antimicrobial spectrum in haloarchaea, as it can inhibit numerous haloarchaeal genera, such as *Halorubrum*, *Haloferax*, *Halobaculum*, *Halobacterium*, *Halopenitus*, and *Haloarcula* (Supplementary Table S1). In addition, strain *Haloferax* sp. Q22 presented no extracellular protease activity (Supplementary Figure S4) but did show an antagonistic effect on strain DF50-ΔEPSΔ*halH4* (Supplementary Figure S5) and other haloarchaeal strains (Supplementary Table S1).

The antagonistic properties of the supernatants of strain *Haloferax* sp. Q22 against strain DF50-ΔEPSΔ*halH4* have been proven successfully (Supplementary Figure S5) indicating that strain DF50-ΔEPSΔ*halH4* does not possess immunity to the halocin(s) from strain *Haloferax* sp. Q22. The antagonistic effect was caused by a proteinaceous substance (Supplementary Figure S6). It was found that high temperature treatment (90°C for more than 10 min) led to inactivation (Supplementary Figure S7), which also indicates that strain *Haloferax* sp. Q22 produces a proteinaceous substance with an antagonistic effect, presumably halocin(s) production.

The effluxes after ultrafiltration with a molecular weight cut-off 3,000 Da presented no inhibition activity, while the effluxes coming through a molecular weight cut-off 10,000 Da ultrafiltration membrane presented a clear inhibition zone (Supplementary Figure S8), which indicates that the molecular weight(s) of the halocin(s) produced by strain *Haloferax* sp. Q22 are between 3,000 and 10,000 Da. Halocin(s) in distilled water can keep the majority of the inhibition activity within 2 h, but the inhibition activity vanished after keeping for over 12 h (data not shown). It indicates that desalting will inactivate the inhibition activity of halocin(s) produced by strain *Haloferax* sp. Q22.

### Promotion of DNA Uptake Efficiency by Halocin(s)

To detect the effect of supernatants of halocin-producing strain on promotion of DNA uptake efficiency, cells of strain DF50-ΔEPSΔ*halH4* were treated with supernatants of strain *Haloferax* sp. Q22 prior to performing the transformation. The efficiency of DNA uptake was significantly increased when the cells of strain DF50-ΔEPSΔ*halH4* were treated with original or twofold concentration of supernatants (Table 4). Transformation efficiency decreased sharply when the cells of strain DF50-ΔEPSΔ*halH4* were treated with supernatants exceeding fourfold condensation before conducting the transformation (Table 4). When the cells of strain DF50-ΔEPSΔ*halH4* were treated with tenfold condensed supernatants, nearly no transformants were obtained. Original supernatants of strain *Haloferax* sp. Q22 could lyse the cells of strain DF50-ΔEPSΔ*halH4* (Supplementary Figure S5) and this effect was even more pronounced in the higher concentrations (four and tenfold).

**TABLE 4:** DNA uptake efficiency of strain DF50-ΔEPSΔ*halH4* treated with the supernatants of strain *Haloferax* sp. Q22.

**Condensational folds of the supernatants**	**DNA uptake efficiency (×10^3^ transformants/μg dsDNA)**
0^∗^	0.002 ± 0.002
1	1.5 ± 0.3
2	4.3 ± 0.4
4	0.035 ± 0.003
10	0

*^∗^Treated with culture medium instead of equal volume of condensed or original (onefold) supernatants. Cells of strain DF50-ΔEPSΔ*halH4* were treated with different condensation of supernatants of strain *Haloferax* sp. Q22 for 1 h prior to performing the simulation natural transformation. Three biological repeats and present the average value.*

### Cell Surface Ultrastructure of Strains DF50, DF50-ΔEPS, DF50-ΔEPSΔ*halH4*, and DF50-ΔEPSΔ*halH4::H4*

**FIGURE 2:**
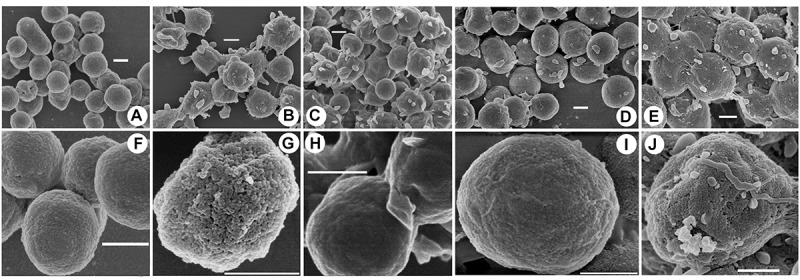
Cell surface uncovered by scanning electron microscopy. Cells of strains were harvested in the late exponential phase by centrifugation. The cell surface ultrastructure of the cells of strains DF50 **(A,F)**, DF50-ΔEPS **(B,G)**, DF50-ΔEPSΔ*halH4* **(C,H)**, DF50-ΔEPSΔ*halH4::H4* **(D,I)**, and DF50-ΔEPSΔ*halH4* treated with original halocin-containing supernatants produced by strain *Haloferax* sp. Q22 **(E,J)** were determined by scanning electron microscopy (HITACHI SU8010, Japan). Bar, 500 nm.

To investigate the potential mechanisms of halocin H4 in DNA uptake, the cell surface ultrastructure of the DF50, DF50-ΔEPS, DF50-ΔEPSΔ*halH4*, DF50-ΔEPSΔ*halH4::H4*, and DF50-ΔEPSΔ*halH4* treated with original halocin produced by strain *Haloferax* sp. Q22 was analyzed. The ultrastructure of the cell surface of strains DF50 and DF50-ΔEPSΔ*halH4* was relatively smooth, while strains DF50-ΔEPS, DF50-ΔEPSΔ*halH4::H4*, and DF50-ΔEPSΔ*halH4* treated with original halocin were rough (Figure 2). When the *halH4* was deleted from strain DF50-ΔEPS resulting in strain DF50-ΔEPSΔ*halH4*, the cell surface was changed from rough to smooth (Figure 2). However, when the *halH4* was introduced back resulting in strain DF50-ΔEPSΔ*halH4::H4*, the cell surface became rougher. It is likely that when cells of strain DF50-ΔEPSΔ*halH4* were treated with original halocin, and the cell surface was changed from smooth to rough in a manner similar to strain DF50-ΔEPS. These results showed that the *halH4* and extracellular halocin played similar roles in changing the ultrastructure of the cell surface. In addition, numerous pinholes (approximately 10 nm in diameter) were present on the cell surface of the strain DF50-ΔEPS or strain DF50-ΔEPSΔ*halH4::H4*, while they were hardly observed on that of the strains DF50 or DF50-ΔEPSΔ*halH4* (Figure 2).

## Discussion

It has been reported that some prokaryotic cells can form a physiological status of natural competence in the logarithmic growth phase, at the transition period between the exponential and stationary phases, or at the onset of the stationary phases (Palmen et al., 1994; Johnsborg et al., 2007). During this period, the cells are prone to take DNA from external sources. Considering the overlap of the reported natural competence formation period, cells of *Hfx. mediterranei* at late exponential phase or at early stationary phase have been used for performing the simulation of DNA uptake in haloarchaea. Previously, we learned that the transcriptional level of *halH4* climbs to its highest point and plateaus at the transition period between the exponential and stationary phases (Cheung et al., 1997). During this period, the transcriptional level of numerous genes including the *halH4* is altered which may lead to the transition of growth phase. Halocins produced by halophilic archaea are antimicrobial peptides or proteins, which generally inhibit the growth of its closely related species (Atanasova et al., 2013). Halocin H4 produced by *Hfx. mediterranei* R4 (=ATCC 33500) is the first halocin that has been studied in depth (Meseguer and Rodriguez-Valera, 1985). In the present study, the correlation between the production of halocin H4 and DNA uptake has been experimentally determined.

Formerly, to explore the biological function, including the inhibition activity of halocin H4, Naor et al. (2013) constructed a *halH4* deletion mutant for further analysis. It was noteworthy that the genetic backgrounds of the *halH4* deletion mutant strains in Naor et al. (2013) and in this study were different. The strains used in Naor et al. (2013) are *pyrE*^–^ and *eps*^+^ (producing EPS), while here the DF50-ΔEPSΔ*halH4* strain was *pyrF*^–^ and defective in EPS production. However, *halH4* deletion mutants in both Naor et al. (2013) and this study maintained their inhibition activity against the sensitive strain *Halobacterium salinarum* NRC1. This suggested that halocin H4 is not the only antimicrobial agent in *Hfx. mediterranei*.

Halocin H6/H7 has been known to inhibit the Na^+^/H^+^ antiporter located on the plasma membrane of the sensitive cells (Meseguer et al., 1995), significantly affecting cell permeability. Halocin C8 has been reported to change the shape of the sensitive cells from rod shaped to spherical, resulting in cell lysis (Li et al., 2003). Cells exposed to their own halocin(s) or to exogenous halocin(s) are probably very similar; our results showed that the halocins (halocin H4 or halocin(s) produced by strain *Haloferax* sp. Q22) trigger a change in the cell envelope ultrastructure (DF50-ΔEPS strain), namely by generating numerous pinholes on the cell surface (Figure 2). The presence of pinholes creates a rough cell membrane ultrastructure in DF50-ΔEPS cells, as opposed to the smooth ultrastructure seen in DF50-ΔEPSΔ*halH4* cells (Figure 2). Compared with strain DF50-ΔEPS, the cell surface of DF50 strain was relatively smooth, a potential result of its EPS production (Figure 2). Halocin H4 may increase the cell permeability but not lead to host cell lysis, thus it is likely a main factor attributed to the natural DNA uptake of *Hfx. mediterranei.* This suggested that a low level of halocin(s) may play a critical role in DNA uptake, but a high level of halocin(s) will lyse the cell.

Competence activators in bacteria have been discussed in the review by Attaiech and Charpentier (2017). Bacteriocins, bacterial protein and peptide antibiotics can promote the uptake of environmental DNA in *B. subtilis*, *Streptococcus mutans*, and other bacteria (van der Ploeg, 2005). A heptadecapeptide pheromone has been used to induce competence in *Streptococcus pneumoniae* (Håvarstein et al., 1995). However, the involvement of halocin(s) in DNA uptake has never been reported. In this study, we found that the deletion of the *halH4* gene tremendously decreased the DNA uptake efficiency in strain DF50-ΔEPSΔ*halH4* (Figure 1 and Table 3) and altered the cell surface ultrastructure, eliminating the presence of pinholes (Figure 2). Original and twofold condensed supernatants of the halocin-producing strain *Haloferax* sp. Q22 could significantly promote the DNA uptake efficiency (Table 4). It is well known that a high concentration of halocin(s) will lyse the cells, which may be attributed to the decrease of the DNA uptake efficiency in treatment with four and tenfold condensed supernatants (Table 4). As *halH4* gene has been well characterized as the encoding gene of the halocin H4, we proposed that halocin H4 would increase the cell membrane permeability, likely by generating pinholes on the cell envelope (directly or indirectly), which may be a prerequisite for inducing DNA uptake in *Hfx. mediterranei*.

**FIGURE 3:**
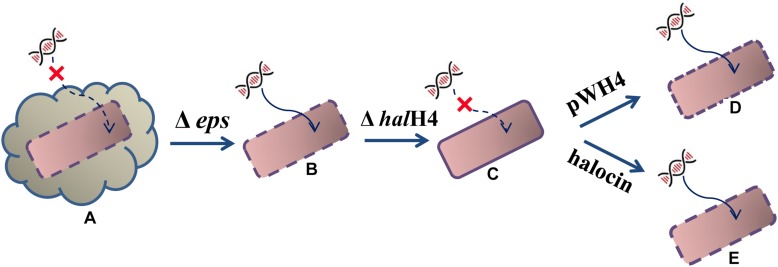
Schematic diagram of halocin involved in DNA uptake. The starting strain DF50 **(A)** exhibiting an exopolysaccharide envelope and a smooth cell surface, are inefficient in taking up external DNA even though the *hal*H4 is active. When the exopolysaccharides envelope was removed resulting in the strain DF50-ΔEPS **(B)**, the cell surface roughened, and they were prone to taking up external DNA. Then, the *hal*H4 was removed resulting in strain DF50-ΔEPSΔ*halH4* **(C)**, the cell surface changed to smooth, and the entry of the external DNA was blocked. If the *hal*H4 was introduced back the strain DF50-ΔEPSΔ*halH4* with plasmid pWH4 resulting in strain DF50-ΔEPSΔ*halH4::H4* **(D)**, the cell surface presented rougher, and they were inclined to take external DNA. When the strain DF50-ΔEPSΔ*halH4* was treated with halocin **(E)**, the cell surface recovered to rough, and external DNA can enter into the cells easily.

Based on the DNA uptake efficiency and cell surface ultrastructure, we proposed a possible hypothesis. The external DNA cannot easily approach the cells of *Hfx. mediterranei* strain DF50 (smooth cell surface) because of the production of exopolysaccharides (EPS). When a gene cluster responsible for exopolysaccharides synthesis was deleted, resulting in strain DF50-ΔEPS (rough cell surface), the external DNA becomes prone to getting into recipient cells. Then, when the *halH4* was deleted resulting in strain DF50-ΔEPSΔ*halH4* (smooth cell surface), the entry of the external DNA was totally blocked. Then, when the *halH4* was introduced back to the *halH4* deletion mutant via a plasmid vector resulting in strain DF50-ΔEPSΔ*halH4::H4* (cell surface becomes rougher than that of strain DF50-ΔEPSΔ*halH4*), or the *halH4* deletion mutant treated with halocin(s) (cell surface similar to that of strain DF50-ΔEPS), the cells regained the ability of taking up external DNA (Figure 3). Here, we concluded that halocin H4, known as a kind of proteinaceous antibiotics, was also involved in inducing DNA uptake in halophilic archaea. To the best of our knowledge, this is the first work uncovering the involvement of halocin H4 in inducing DNA uptake (potentially as a competence activator or inducer) in halophilic archaea.

## Data Availability

The raw data supporting the conclusions of this manuscript will be made available by the authors, without undue reservation, to any qualified researcher.

## Author Contributions

SC and HX designed the experiments and analyzed the data. SC, SS, and JL performed the experiments. SC and GK wrote the manuscript. HX and GK proofread the manuscript.

## Conflict of Interest Statement

The authors declare that the research was conducted in the absence of any commercial or financial relationships that could be construed as a potential conflict of interest.

## Footnotes

1 http://primer3.ut.ee/
