# Supplementary material for: A Halocin Promotes DNA Uptake in Haloferax mediterranei
Source: Front Microbiol. 2019 Sep 18;10:1960. doi: 10.3389/fmicb.2019.01960 (PMC6759562; doi:10.3389/fmicb.2019.01960)
Supplement: Supplementary file 1 [file Data_Sheet_1.doc]

**Supplementary materials**

**Table S1. Detection of inhibition effect of strain *Haloferax*** sp. Q22 to partial of haloarchaeal strains.

| Indicator strain | Inhibition activity | Indicator strain | Inhibition activity |
| --- | --- | --- | --- |
| *Halorubrum* *salinum* GX71 | + | *Halobacterium salinarum* NRC-1 | + |
| *Halorubrum saccharovorum* CGMCC 1.2147 | + | *Halobacterium noricense* A1 | + |
| *Halorubrum aidingense* 1.2670 | + | *Halococcus salsus* ZJ1 | – |
| *Halorubrum halophilum* JCM 18963 | + | *Halococcus hamelinensis* JCM 12892 | – |
| *Halorubrum trueperi* Y73 | + | *Halopenitus malekzadehii* CC65 | + |
| *Halorubrum lipolyticum* 9-3 | + | *Haloarcula amylolytica* BD-3 | + |
| *Halorubrum pallidum* PJ61 |  | *Haloarcula argentinensis* arg-1 | + |
| *Haloferax volcanii* DS2 | + | *Haloparvum sediment* DYS4 | + |
| *Halobaculum roseum* D90 | + | *Halalkalicoccus subterraneus* GSM28 | – |
| *Halobaculum gomorrense* JCM 9908 | + | *Halalkalicoccus tibetensis* CGMCC 1.3240 | – |

“+” antagonistic effect; “–” no inhibition effect.

**
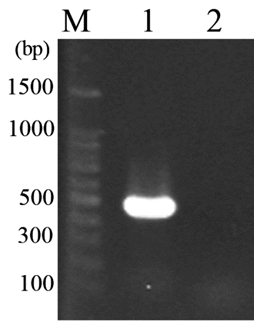
**

**Fig. S1 PCR verification of the deletion of the *halH4*.** Cells of strains DF50-ΔEPS and DF50-ΔEPSΔ*halH4* lysed in distilled water were taken as PCR template. Pair of primers H4F and H4R (Table 2) was taken as PCR primers. PCR products of strains DF50-ΔEPS (lane 1) and DF50-ΔEPSΔ*halH4* (lane 2) were loaded to the sample holes (1% agarose) for electrophoresis detection. M, 100 bp DNA ladder. The sizes of DNA bands are shown on the left.


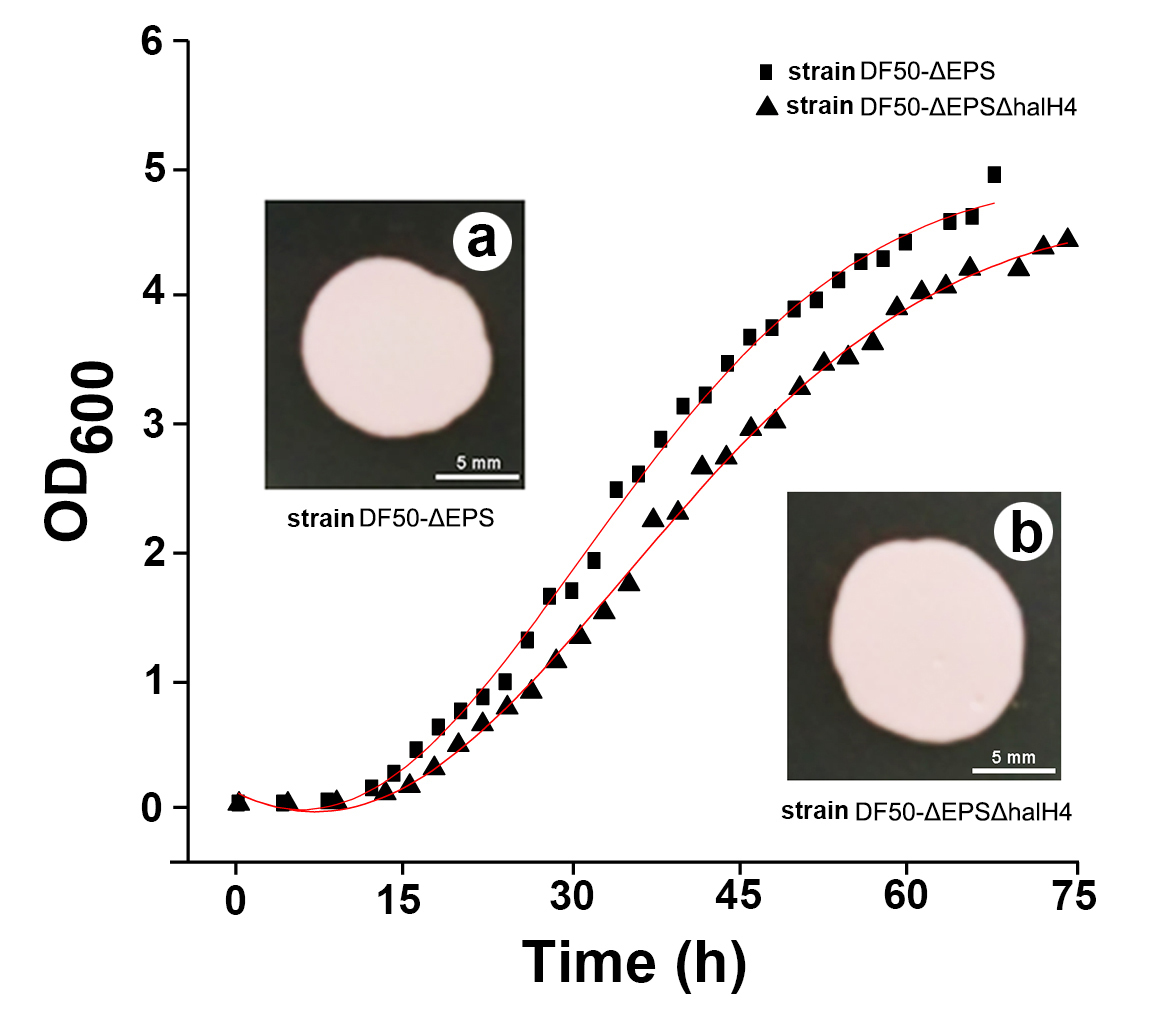


**Fig. S2 Growth in liquid medium and on agar plate of the strains DF50-ΔEPS and DF50-ΔEPSΔ*halH4*.** Strains of DF50-ΔEPS and DF50-ΔEPSΔ*halH4* were cultured in liquid AS-168 medium supplemented with uracil, and then 1 mL of each cell suspension was inoculated into the flask containing 100 mL liquid medium at 37 °C with shaking at 180 rpm. The optical density at 600 nm was recorded at an interval of 4 h. After 12 h, the interval of recording was adjusted to 2 h. Then, 10 μL of each cell suspension of strains DF50-ΔEPS (a) and DF50-ΔEPSΔ*halH4* (b) were dropped onto solid AS-168 plate supplemented with uracil. After incubation at 37 °C for 7 days, photographs were taken.


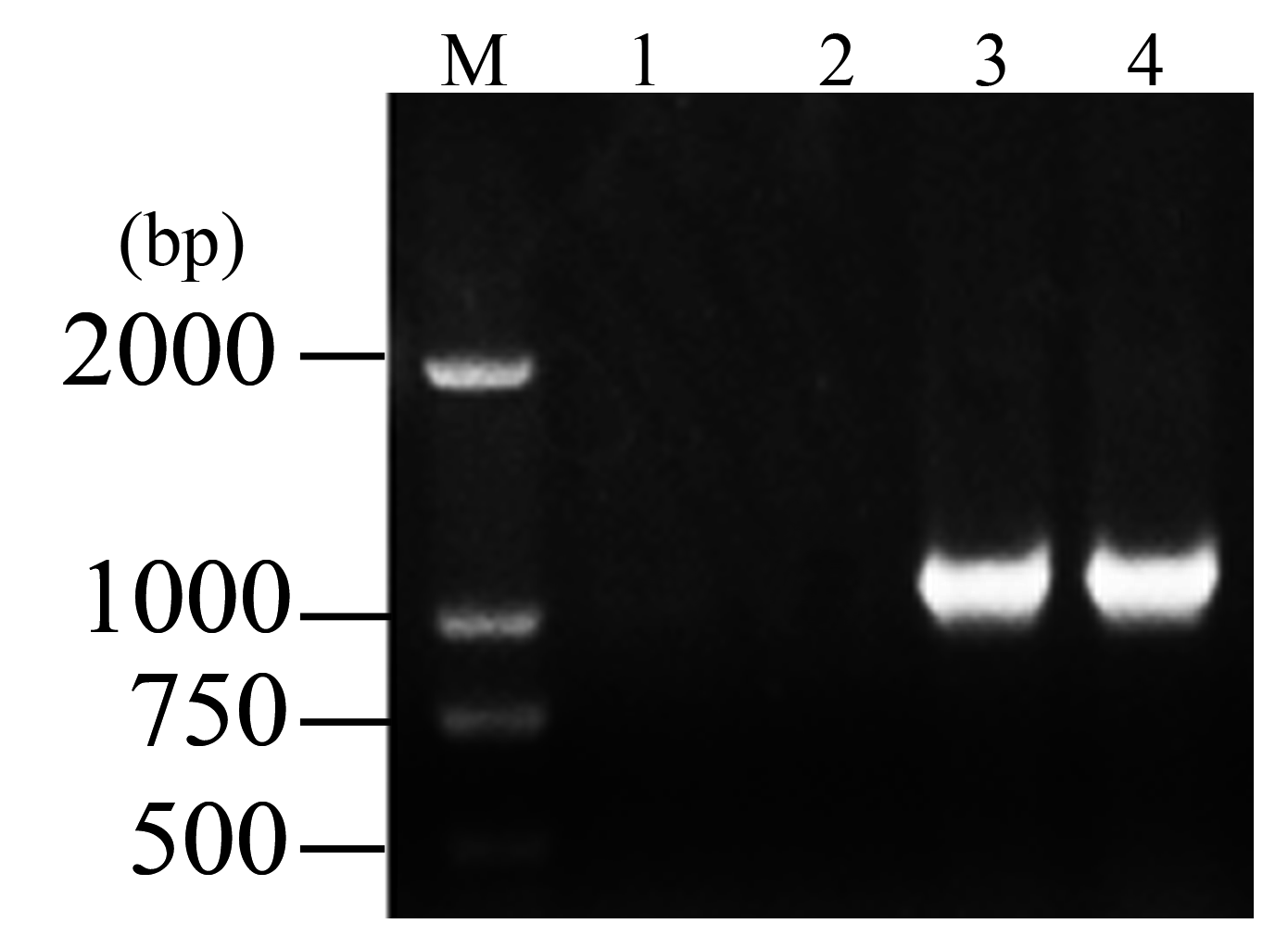


**Fig. S3 PCR verification of the *halH4* complementary strain.** Cells of strains DF50-ΔEPSΔ*halH4* and DF50-ΔEPSΔ*halH4::H4* lysed in distilled water were taken as PCR template. Pair of primers H4CF and H4CR (Table 2) was used. PCR products of strains DF50-ΔEPSΔ*halH4* (lane 1 and 2) and DF50-ΔEPSΔ*halH4::H4* (lane 3 and 4) were loaded to the sample holes (1% agarose) for electrophoresis detection. M, DNA ladder; the sizes of DNA bands are shown on the left.


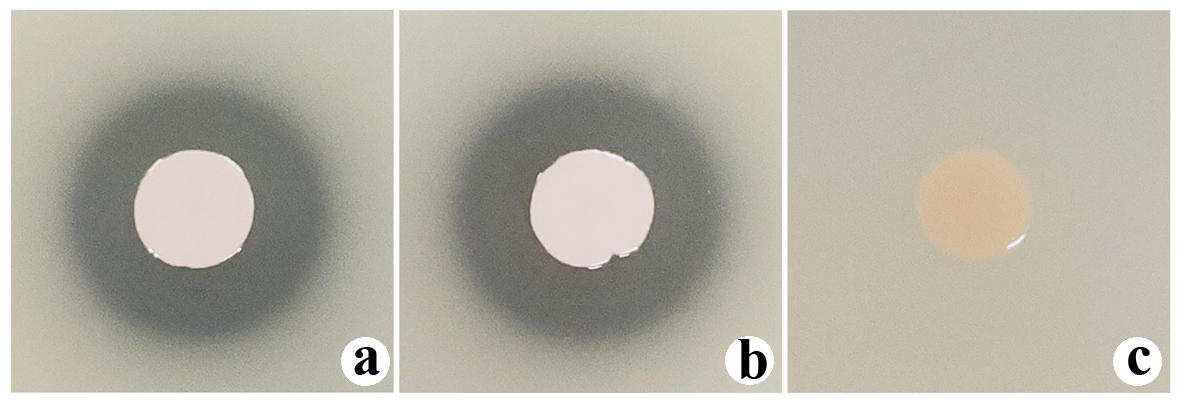


**Fig. S4 Detection of the extracellaluar protease activity.** Cells of strains DF50-ΔEPS (a), DF50-ΔEPSΔ*halH4* (b) and *Haloferax* sp. Q22 (c) grown on skim milk agar plates.


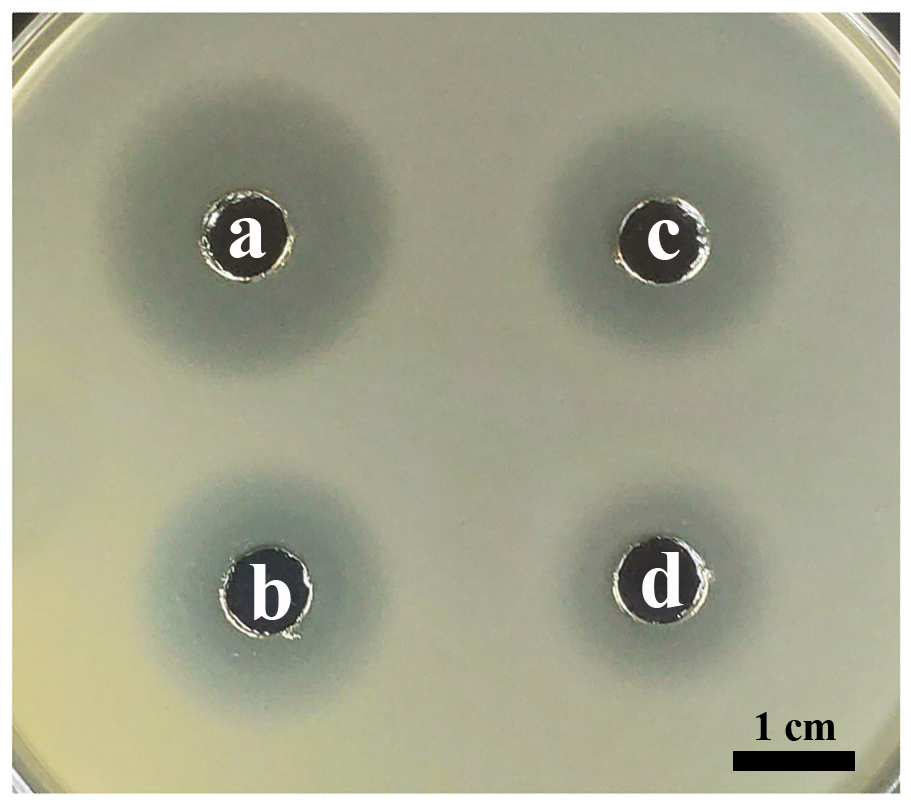


**Fig. S5 Supernatants of *Haloferax* sp. strain Q22 inhibiting the growth of strain DF50-ΔEPSΔ*halH4*.** The diameter of inhibition zones represents inhibiting effect of supernatants of *Haloferax* sp. strain Q22 against strain DF50-ΔEPSΔ*halH4*. Original supernatants of *Haloferax* sp. strain Q22 were prepared by removing the cells using centrifugation (12,000 g, 3 min) followed by filtration (pore size, 0.22 μm). 100 μl (a), 80 μl (b), 60 μl (c) and 40 μl (d) of supernatants were added to the holes, and then cultivated at 37 °C for 2 days. Diameter of the hole for pouring the supernatants of *Haloferax* sp. strain Q22 is 6 mm. Bar, 1 cm.


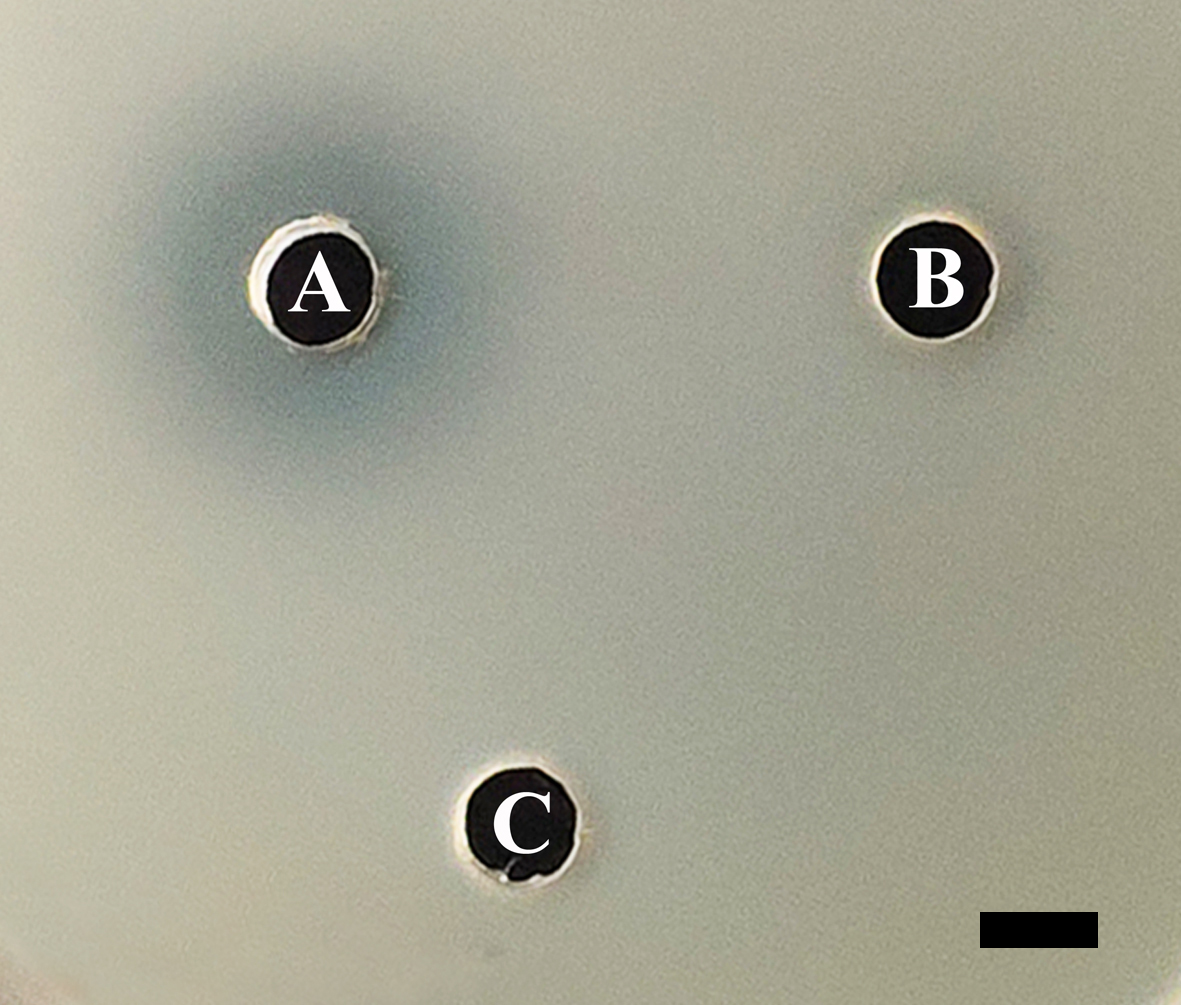


**Fig. S6** **Protease K eliminates the inhibition effect of the** **supernatants of *Haloferax* sp. Q22 against strain DF50-ΔEPSΔ*halH4*.** Strain DF50-ΔEPSΔ*halH4* was taken as the indicator. One hundred microliters of the original supernatants of *Haloferax* sp. Q22 (A), original supernatants treated with protease K (B), and protease K (C) solution in AS-168 medium were poured in corresponding holes. The final concentration of protease K is set to 5 mg/ml. Bar, 6 mm.


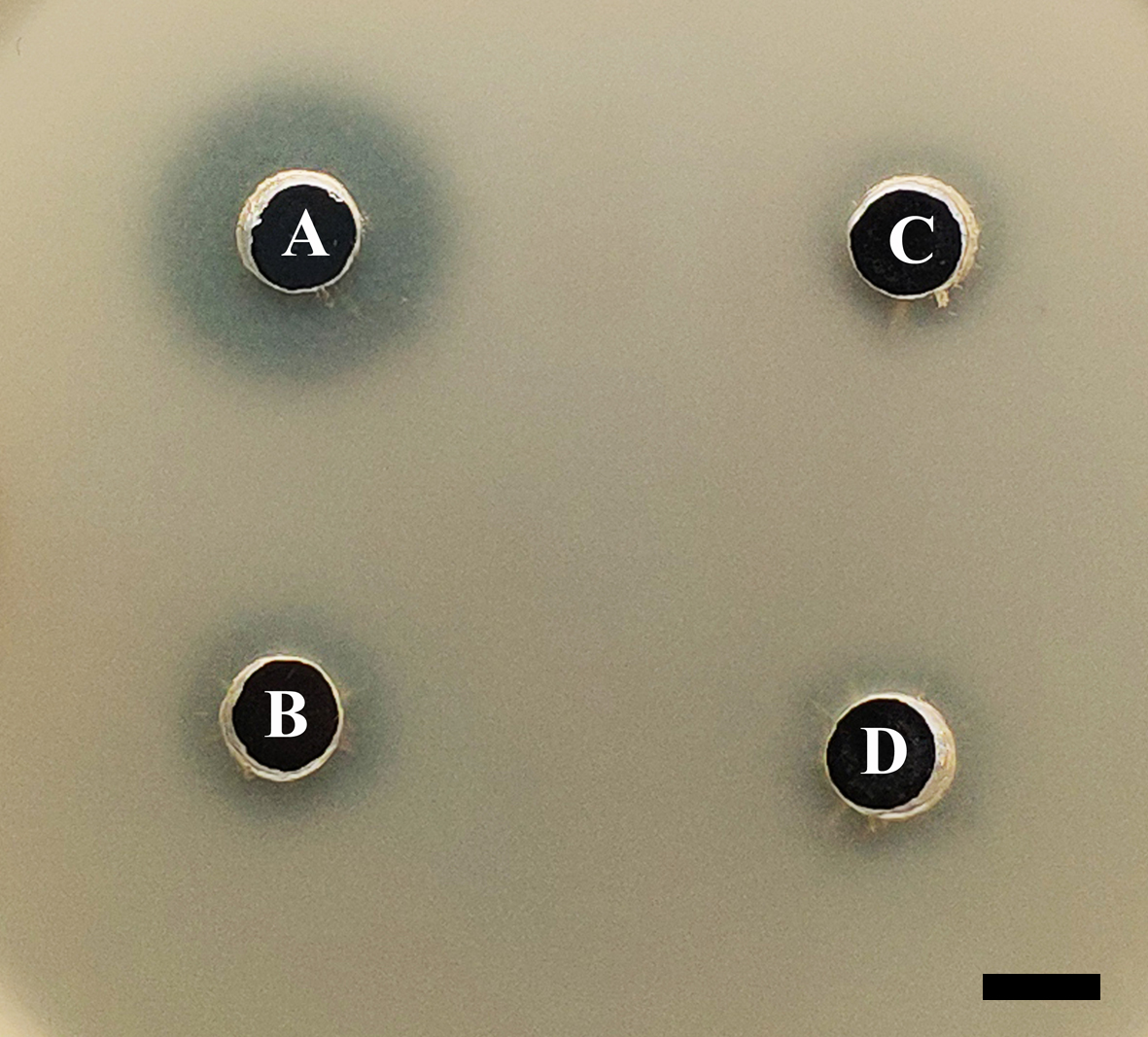


**Fig. S7 Thermostability of the** **supernatants of *Haloferax* sp. Q22.** Strain DF50-ΔEPSΔ*halH4* was taken as the indicator. One hundred microliters of the original supernatants of *Haloferax* sp. Q22 (A) and heat treated (B, C and D) supernatants were used. Supernatants of *Haloferax* sp. Q22 were heated at 90 °C for 10 min (B), 30 min (C) and 60 min (D) respectively before pouring to the hole. Bar, 6 mm.


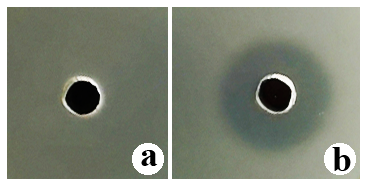


**Fig. S8 Determination of the molecular weight of the** **halocin(s) produced by steain *Haloferax* sp. Q22.** 100 μL of the effluxes after ultrafiltration with a molecular weight cut-off 3000 Da (a) and 10,000 Da (b) were dropped to the holes on indicator plate. Strain DF50-ΔEPSΔhalH4 was used to construct the indicator plate. Diameter of the hole is 6 mm.
